# “Health in the Mirror”: An Unconventional Approach to Unmet Psychological Needs in Oncology

**DOI:** 10.3389/fpsyg.2017.01633

**Published:** 2017-09-21

**Authors:** Valentina E. Di Mattei, Letizia Carnelli, Paola Taranto, Martina Bernardi, Chiara Brombin, Federica Cugnata, Angela Noviello, Morag Currin, Giorgia Mangili, Emanuela Rabaiotti, Lucio Sarno, Massimo Candiani

**Affiliations:** ^1^Faculty of Psychology, Vita-Salute San Raffaele University Milan, Italy; ^2^Clinical and Health Psychology Unit, Department of Clinical Neurosciences, IRCCS San Raffaele Hospital Milan, Italy; ^3^Languages Department, University of Parma Parma, Italy; ^4^University Centre of Statistics in the Biomedical Sciences, Vita-Salute San Raffaele University Milan, Italy; ^5^Oncology Training International Balgonie, SK, Canada; ^6^Department of Obstetrics and Gynecology, IRCCS San Raffaele Hospital Milan, Italy; ^7^Faculty of Medicine, Vita-Salute San Raffaele University Milan, Italy

**Keywords:** supportive care, complementary therapy, cancer, women, oncology aesthetics, Health in the Mirror, Italy

## Abstract

**Background:** The introduction of aesthetic care programs for cancer patients inside hospitals could help patients cope with the side effects of both disease and treatment. The specific objective of this study is to evaluate whether a complementary and supportive program, called “Health in the Mirror,” has a positive effect on participants by analyzing certain psychological variables.

**Methods:** Eighty-eight female cancer patients were included in this analysis. The support program is composed of three group aesthetic interventions that address both physical and psychological aspects that accompany cancer and its treatment. Patients were asked to complete a battery of tests in order to measure the impact of the program on certain psychological variables including anxiety, depression, body image, self-esteem, and quality of life. Outcome variables were measured at three different time-points: prior to participation, on the last day of the program, and after a 3-month follow-up.

**Results:** Participating in the psychosocial support program “Health in the Mirror” determines an improvement in the psychological variables measured. Results revealed a significant reduction in depressive symptoms, anxiety and body image issues, as well as an improvement in self-esteem levels; this suggests that participating in this program could facilitate better adjustment to disease and treatment.

**Discussion:** This study legitimizes the importance of implementing supportive and complementary therapies together with conventional therapies; the therapeutic approach to cancer cannot be restricted solely to medical care, but it must consider the patient as a whole person with needs that are not only physical or medical, but also psychological, social, and existential.

## Introduction

According to the Italian Association of Cancer Registries, it is estimated that in 2014 around 2,250,000 people in Italy were living with a preceding cancer diagnosis. Among these, 56% were women.

Women are affected physically and psychologically after a cancer diagnosis and the beginning of treatment; these processes can often determine the onset of anxious and depressive symptoms (Linden et al., 2012; Jones et al., 2015; Stafford et al., 2015) and have a negative influence on quality of life (Montazeri et al., 2008; Reis et al., 2010; Jones et al., 2015), sexual functioning, self-esteem, and body image (Fobair et al., 2006; Reis et al., 2010; Pinar et al., 2012; Rosenberg et al., 2013). Despite recent improvements in cancer treatments, there are still severe side effects, such as nausea, vomiting, appetite loss, fatigue, alopecia and all the consequences of corticosteroid treatment (e.g., difficulty sleeping and weight gain) (Carelle et al., 2002). Patients often report all these side effects as distressing as the diagnosis itself; in particular, aesthetic side effects, specifically hair loss, are often reported by women as the most disturbing and traumatizing (Lemieux et al., 2008; Kim et al., 2012).

To address the need to care for patients' quality of life and wellbeing, complementary therapies are being increasingly implemented alongside conventional medical therapies, toward an integrative model of medical care. These adjunctive therapies include mind-body techniques, such as meditation, guided imagery and expressive arts (music and art therapy), nutrition and nutritional supplements, physical activity and exercise, acupuncture, massage therapy, and energy therapies (Deng et al., 2009).

The Academic Consortium for Integrative Medicine and Health defines integrative medicine as:

*the practice of medicine that reaffirms the importance of the relationship between practitioner and patient, focuses on the whole person, is informed by evidence, and makes use of all appropriate therapeutic approaches, healthcare professionals and disciplines to achieve optimal health and healing* (Kligler et al., 2004 pp. 522).

Within this framework, introducing aesthetic care programs for cancer patients inside hospitals could help women cope with the side effects of both disease and treatments that can affect their body image. Up to now, few studies have investigated the efficacy of these programs on women's wellbeing, both with the aid of qualitative (Amiel et al., 2009; Zannini et al., 2012) and quantitative data (Quintard and Lakdja, 2008; Taggart et al., 2009). The literature indicates that participating in aesthetic care programs could determine an improvement in body image, anxiety, self-esteem, and the quality of social interactions (Quintard and Lakdja, 2008; Taggart et al., 2009), thus facilitating a better adjustment to disease.

“Health in the Mirror” is a psychosocial support program for female cancer patients which was developed in 2013 at a Hospital in Northern Italy. It is aimed at helping patients manage appearance-related side effects resulting from cancer treatment, thus promoting their wellbeing, quality of life, and adjustment to illness.

In the framework of clinical management of oncological disease, “Health in the Mirror” represents an answer to the need to gain a more global vision of patient medical care, not just by “curing” the disease, but also by “taking care” of the person as a whole, by addressing quality of life, distress symptoms, and personal well-being. The specific objective was to evaluate whether the “Health in the Mirror” program has a positive effect on participants by analyzing whether participation determines an improvement in certain psychological variables including depression, anxiety, self-esteem, body image, and quality of life. Our first hypothesis is that after participating in the program, participants scores would improve on all psychological aspects investigated in the study. Furthermore, we hypothesized that these effects would persist for at least 3 months after participation in the “Health in the Mirror” program.

## Materials and methods

### Sample selection and recruitment

After an initial preliminary study (Di Mattei et al., 2014), where results were promising, a sample of patients was recruited from the Gynecology and Oncology Units at a Hospital in Northern Italy. Eligibility criteria included: (1) being a female patient; (2) having a cancer diagnosis and undergoing chemotherapy treatment; (3) not being in a clinical condition that would make it unsafe to participate in a group context (e.g., severe neutropenia); (4) being able to read and understand Italian; (5) being at least 18 years old; and (6) agreeing to voluntarily participate in the program. Furthermore, each patient's medical team gave authorization for the patient to take part in the program.

A total of 114 patients participated in our program; only those who completed both the baseline and follow-up surveys were included (*n* = 88). The discrepancy between the number of participants at t_0_ (*n* = 114) and the number of participants at t_2_ (*n* = 88) is due to the exclusion of 16 patients who did not attend all of the sessions and 10 patients who unfortunately passed away during the study period.

### Procedure

All procedures performed involving human participants were conducted in accordance with the ethical standards of the IRCCS San Raffaele Hospital research committee and with the 1964 Helsinki declaration and its later amendments or comparable ethical standards. The study was approved by the Medical Ethical Committee of the IRCCS San Raffaele Hospital on October 8th, 2015. Participants provided their written informed consent to take part in the study and patient anonymity was guaranteed. Participants were told that they could withdraw from participation at any time if they wished to. The study data was collected between October 2015 and October 2016.

After explaining the purpose of the research and obtaining informed consent, we asked participants to complete a battery of tests composed of five self-report questionnaires. The battery was administered at three time points: 2 weeks prior to participating in the program, during a preliminary interview with a psychologist (t_0_), at the end of the last session of “Health in the Mirror” (t_1_), and at a 3-month follow-up after participation (t_2_).

The program is composed of an initial psychological assessment and three group sessions that take place on a weekly basis; groups are usually composed of 10-12 patients. During the first session, both a make-up artist and wig expert illustrate strategies to manage the aesthetic side effects of cancer treatment that may affect one's appearance, such as pale skin color and hair loss, including the loss of eyelashes and eyebrows. After an initial make-up and wig tutorial, each patient receives a personalized make-up session and is given the possibility to choose a wig to keep. A photographer is also present during the day, in order to capture patients' portraits before and after their personalized treatment. The second session is divided into an educational and a practical tutorial. During the morning, an esthetician trained in oncology aesthetics (according to the Oncology Training International guidelines) gives a lecture on how to treat the skin and body during cancer treatments (e.g., cosmetic ingredients to avoid and how to moisturize and cleanse on a daily basis). During the afternoon, a prestigious spa is recreated within the hospital, where patients can receive aesthetic treatments (e.g., massages, manicures, and pedicures). These treatments are all carried out by experts trained in oncology aesthetics who take each patient's condition in consideration during the beauty treatments. Moreover, during the afternoon, the fashion stylist gives a personalized consultation on the use of colors to match each patient's skin tone. The last session is dedicated to a group discussion, led by a team of psychologists, where patients can share their thoughts and feelings regarding their cancer experience and their experience of participating in the Health in the Mirror program. Throughout the sessions, psychologists and oncologists are always present, as taking part in this project is considered an integral part of a patient's treatment, while continuing medical treatments concurrently. The presence of psychologists also gives patients the opportunity to confront any difficulties or feelings, in a timely manner, that may arise during a group situation such as this.

### Measures

Patient socio-demographic (age, marital status, presence of children, and profession) and clinical characteristics (type of cancer diagnosis, date of diagnosis, presence/absence of relapse/metastases, type of treatment received, presence/absence of cancer family history, previous/present psychological interventions, and use of psychotropic medication) were collected via a self-report questionnaire and via access to medical records.

Quality of life was assessed via the European Organization for Research and Treatment of Cancer Study Group QLQ-C30 (EORTC QLQ-C30) (Aaronson et al., 1993). This questionnaire is composed of 30 items and includes a global health and quality of life scale, five functional subscales evaluating physical, role, emotional, cognitive, and social functioning and three symptom subscales evaluating nausea and vomiting, pain, and fatigue. Six single items assess financial difficulties and symptoms that are frequently reported by cancer patients (dyspnea, insomnia, appetite loss, constipation and diarrhea). Responses are given on a 4-point Likert Scale from 1 (not at all) to 4 (very much), with total scores ranging from 0 to 100; higher scores correspond to a better level of functioning for the functional and global scales and to a higher severity of symptoms for the symptom scales. The EORTC QLQ-C30 has shown good reliability (Cronbach's α ranges from 0.54 to 0.86 before treatment and from 0.52 to 0.89 after treatment) and clinical validity (Aaronson et al., 1993).

Levels of depressive symptoms were assessed using the Beck Depression Inventory-II (BDI-II) (Beck et al., 1996), a widely-used measure to evaluate the presence of depression criteria according to the DSM-IV. It consists of 21 items divided into two subscales: Cognitive-Affective and Somatic-Vegetative (Dozois et al., 1998); each item includes four response options arranged in increasing severity (0 = absent to 3 = severe). Scoring is obtained by summing up all of the item scores; different ranges of severity have been defined on an empirical basis, 0–13 = minimal depression, 14–19 = mild depression, 20–28 = moderate depression, and 29–63 = severe depression (Dozois et al., 1998). Cronbach's α scores for the BDI-II range from 0.92 to 0.93 and convergent and discriminant validity are consistent (Beck et al., 1996); values of internal consistency for the Italian version range from 0.80 to 0.87 (Ghisi et al., 2006).

Anxiety levels were evaluated via the State-Trait Anxiety Inventory—Y Form (STAI-Y) (Spielberger et al., 1983). This self-report measure consists of two subscales including 20 items each: the state subscale measures anxiety at the moment of questionnaire completion and is administered first, whereas the trait subscale refers to how a person “usually” feels on a day-to-day basis. Responses are reported on a 4-point Likert scale from 1 (not at all) to 4 (very much). Total scores range from 20 to 80 and can be grouped as follows: 20–39 = low anxiety, 40–59 = medium anxiety, 60–80 = high anxiety. The STAI has demonstrated good reliability (Cronbach's α.83– 0.95) and validity (Spielberger et al., 1983). The Italian version of the STAI-Y was validated by Pedrabissi and Santinello (1989).

The Body Image Scale (BIS) (Hopwood et al., 2001) measures perceived body image in cancer patients. It is a 10-item scale including affective items, behavioral items, and cognitive items. Response options vary from 0 (not at all) to 3 (very much), with a maximum total score of 30; higher scores correspond to more body image concerns. Reliability coefficients range from 0.86 to 0.93; the scale has also demonstrated good clinical validity, both in the original (Hopwood et al., 2001) and the Italian version (Cheli et al., 2016).

Self-esteem levels were assessed using the Rosenberg Self-Esteem Scale (RSE) (Rosenberg, 1965), a quick, easy-to-understand and internationally used measure of self-esteem. It is composed of 10 items, which ask patients to evaluate their degree of agreement with each statement; there are four options of response (1 = strongly disagree to 4 = strongly agree). Five items are presented positively; the other five are reverse scored. Total scores range from 11 to 40, where higher scores indicate higher levels of self-esteem. The Italian version of the RSE has demonstrated good reliability (Cronbach's α of 0.84) and clinical validity (Prezza et al., 1997).

### Statistical methods

Continuous variables have been reported as mean, range, and standard deviation, while for categorical variables have been described in terms of frequency distribution. Psychometric variables of interest, collected over time, have been modeled using suitable techniques for repeated measures data.

Linear Mixed Effects (LME) models (Laird and Ware, 1982; Pinheiro and Bates, 2000) are commonly applied to model data gathered over time. By allowing the specification of random components (e.g., subject or time specific random effects), these models provide an appropriate framework for representing longitudinal data, accounting for unobserved heterogeneity. However, LMEs are suited for Gaussian response variables and assume a linear link between covariates and outcome. These assumptions are not always met, especially in psychological studies, usually dealing with ordinal/non-Gaussian/skewed psychometric scales.

In these cases, an extension of standard LME should be considered. In particular, as proposed in Proust-Lima et al. (2015), we chose a flexible modeling strategy within the latent class mixed model (LCMM) framework.

Let us consider a sample of N subjects measured in time. For each subject, several measurements Y_ij_, i = 1, …, N, j = 1, …., n_i_ of the outcome of interest are available at time t_ij_. Following the literature on latent variable modeling, a latent model, that is, a standard linear mixed model without measurement errors, along with a measurement model, linking the latent process to the outcome of interest, must be specified. The latent process Λ_i_(t) is a function of time-dependent mixed (fixed and random) covariates, defined as,

$$\begin{array}{l} \Lambda_{\text{i}}\;\left(\text{t}\right)\;=\;{\text{X}}_{\text{Li}}{\left(\text{t}\right)}^{\text{T}}\beta \;+\;{\text{Z}}_{\text{i}}{\left(\text{t}\right)}^{\text{T}}{\text{u}}_{\text{i}}\;+\;{\text{w}}_{\text{i}}\left(\text{t}\right),\text{ t }\geq \;0 \end{array}$$

where X_Li_(t) and Z_i_(t) are vectors of time-dependent covariates associated respectively with the vector of fixed effects β and the random effects u_i_ and w_i_(t) represent an auto-regressive process.

Then a measurement model ruling the relationship between the longitudinal outcome Y_ij_, observed at time t_ij_, and the latent process Λ_i_(t) is defined as,

$$\begin{array}{l} {\text{Y}}_{\text{ij}}\;=\text{ H}\left(\Lambda_{\text{i}}\left({\text{t}}_{\text{ij}}\right)+\epsilon_{\text{ij}};\eta \right) \end{array}$$

where H(; η) is a parametrized link function, ϵ_ij_ are independent normally distributed errors.

Depending on the choice of H, curvilinear, ordinal, non-normal longitudinal outcomes can be easily handled. The procedure does not require that subjects are observed at the same time points or have the same number of measurements.

The significance threshold was set equal to 0.05. All analyses were performed using R statistical software version 3.2.5 (R Core Team, 2016) and *lcmm* package (Proust-Lima et al., 2016) was used to estimate latent mixed models.

## Results

### Sample socio-demographic and clinical characteristics

The mean age of the study participants was 50.83 (*SD* = 11.40), ranging from 20–76 years. In Table 1, socio-demographic and clinical characteristics are shown. The majority of patients were married (67%) and had children (77%). The most prevalent cancer diagnoses in our sample were breast (41%) and gynecologic cancers (46%); the remaining diagnoses comprised brain, lung, hematologic, and colorectal cancers. Seventeen patients were experiencing a relapse (19%) and 23 patients had metastases (26%). In addition to chemotherapy, 16% of patients were also receiving radiation therapy and 75% had undergone surgery. The mean time from diagnosis to questionnaire completion was 19.8 months (*SD* = 35.5).

**Table 1:** Socio-demographic and clinical characteristics.

**Characteristic**	**Freq**.	**%**
**MARITAL STATUS**		
Married	59	67.05
Not Married	29	32.95
**PRESENCE OF CHILDREN**		
Yes	68	77.27
No	20	22.73
**DIAGNOSES (*N* = 87)**		
Breast cancer	36	41.38
Gynecologic cancers	40	45.98
Other	11	12.64
**RADIATION THERAPY**		
Yes	14	15.91
No	74	84.09
**RELAPSE**		
Yes	17	19.32
No	71	80.68
**METASTASES**		
Yes	23	26.14
No	65	73.86
**SURGERY**		
No	22	25
Mastectomy	13	14.77
Quadrantectomy	15	17.05
Hysterectomy/oophorectomy	32	36.36
Other	6	6.82
**PREVIOUS PSYCH. INT. (*N* = 87)**		
Yes	29	33.33
No	58	66.67
**PSYCHOTROPIC MED. (*N* = 87)**		
Yes	21	24.14
No	66	75.86

Descriptive statistics of psychometric variables of interest at each of the three time points are shown in Table 2. The mean score on the Beck Depression Scale was 11.23 at the preliminary interview (t_0_), 8.38 at the end of the last session of “Health in the Mirror” (t_1_) and 8.52 at a 3-month follow-up after participation in the program (t_2_). Twelve out of 88 women obtained a score greater or equal to 20, which indicates moderate or severe depression, at the preliminary interview, 3 at t_1_, and 4 at t_2_. The STAI State scale mean was 42.68 at t_0_, 36.66 at t_1_, and 39.66 at t_2_. Twelve women presented high levels of State anxiety (scores ≥ 60) at t_0_. None of the participants showed STAI values above this threshold at t_1_ and at t_2_ six participants had increased STAI values, thus suggesting a worsening in anxiety levels (scores ≥ 60).

**Table 2:** Descriptive statistics of investigated psychometric variables of interest at each time point.

	***n***	**M *(SD)***	**Min**.	**Max**.	Reference values^*^
**t_0_**					
Beck Depression Inventory-II	88	11.23 (7.41)	1	31	7.79 (6.41)
State-Trait Anxiety Inventory - STATE	88	42.68 (12.56)	20	69	41.3 (10.79)
EORTC QLQ-C30–global health status	88	65.72 (20.66)	16.67	100	59.3 (24.9)
Body Image Scale	86	8.94 (6.88)	0	28	7.64 (7.22)
Rosenberg Self-Esteem Scale	88	33.68 (5.13)	19	40	29.09^**^
**t_1_**					
Beck Depression Inventory-II	88	8.38 (5.39)	1	30	7.79 (6.41)
State-Trait Anxiety Inventory - STATE	88	36.66 (9.02)	20	59	41.3 (10.79)
EORTC QLQ-C30–global health status	87	65.23 (18.77)	16.67	100	59.3 (24.9)
Body Image Scale	88	7.03 (5.49)	0	26	7.64 (7.22)
Rosenberg Self-Esteem Scale	88	33.8 (4.5)	20	40	29.09^**^
**t_2_**					
Beck Depression Inventory-II	88	8.52 (6.5)	0	39	7.79 (6.41)
State-Trait Anxiety Inventory - STATE	88	39.66 (10.7)	20	62	41.3 (10.79)
EORTC QLQ-C30 – global health status	88	70.64 (17.78)	25	100	59.3 (24.9)
Body Image Scale	88	6.6 (5.82)	0	22	7.64 (7.22)
Rosenberg Self-Esteem Scale	88	34.78 (5.54)	11	40	29.09^**^

* *Mean and standard deviations of reference values reported for women for BDI-II (Ghisi et al., 2006), STAI-State (Pedrabissi and Santinello, 1989), EORTC QLQ-C30 (Scott et al., 2008), and BIS (Hopwood et al., 2001)*.

** *Average value retrieved from Prezza et al. (1997) not age-specific*.

### Multivariate analyses

Patient socio-demographic characteristics, in particular age and the presence/absence of children, along with several clinical characteristics (cancer diagnosis, presence/absence of relapse, type of (breast) surgery, previous/present psychological interventions and use of psychotropic medication) were included in the model, along with a time variable. The latter was entered as an indicator variable of the three time points (t_0_, t_1_, and t_2_) in which psychometric scales were measured. Nonlinear link functions were chosen (splines transformation with 5 equidistant knots) to normalize the outcome psychometric scales (Ramsay, 1988). The choice of variables to enter into the model was led by considerations on multicollinearity issues. Stepwise backward procedures for model selection were then applied (Table 3).

**Table 3:** Estimates (standard-errors) of the models.

	**BDI**	**STAI**	**EORTC QLQ**	**BIS**	**SE**
t_1_	−0.64 (0.18)^***^	−0.88 (0.20)^***^	−0.08 (0.17)	−0.53 (0.17)^**^	−0.01 (0.15)
t_2_	−0.68 (0.20)^***^	−0.42 (0.17)^*^	0.30 (0.16)	−0.78 (0.22)^***^	0.46 (0.16)^**^
Age	−0.03 (0.01)^*^			−0.05 (0.02)^**^	
Presence of children					0.80 (0.39)^*^
Diagnose (ref = Breast cancer)					
Gynecologic cancers		0.50 (0.28)	−0.06 (0.24)		
Other		0.86 (0.43)^*^	−0.76 (0.37)^*^		
Previous psych. int.		0.55 (0.28)^*^			
Relapse	1.02 (0.40)^*^		−0.80 (0.28)^**^	1.81 (0.48)^***^	−1.36 (0.43)^**^

*** *p < 0.001*,

** *p < 0.01*,

* *p < 0.05*.

BDI values significantly decreased at t_1_ and t_2_ with respect to the baseline values (t_0_). We found that depression significantly decreases with age. Moreover, experiencing a relapse significantly increases depression levels. Similar results were found when examining BIS levels.

When analyzing the STAI, we found that anxiety levels significantly decreased at t_1_ and t_2_ with respect to the t_0_, with a larger decrease in t_1_. The presence of other diagnoses (brain, lung, hematologic, and colorectal cancers), when compared to breast cancer, and previous psychiatric interventions significantly increased anxiety levels.

Global health status, measured via the EORTC scale, was affected by the presence of relapses and the presence of other diagnoses, as opposed to a breast cancer diagnosis. These factors significantly decreased health status. We did not find significant changes over time in EORTC levels.

Finally, self-esteem scores significantly increased at time t_2_ with respect to t_0_. Having children was found to positively affect SE values, while the presence of relapses had a significant and negative effect on this variable.

## Discussion

In spite of the advances in cancer care and survival rates, patients continue to experience invalidating side effects both during and after cancer treatment; these affect patients not only on a physical, but also on a psychological, social, and spiritual level (Grassi et al., 2003). The use of complementary and supportive therapies is becoming increasingly more extensive among cancer patients and cancer survivors, thus more research is required to address the effects these have on global well-being and quality of life.

There is a plethora of literature demonstrating the effectiveness of complementary therapies, such as mind-body techniques, acupuncture, manipulative and body-based practices, that help with pain and symptom management, reduce depression and anxiety, and improve the quality of life of patients during and after treatment (Deng and Cassileth, 2013; Johnson et al., 2014; Frenkel et al., 2015). Moreover, adjunctive therapies such as diet and dietary supplements, physical exercise, and stress reduction techniques may also improve survival and reduce the risk of recurrence (Frenkel et al., 2015).

A cancer diagnosis forces women to confront aggressive treatment side effects and these determine important changes to their physical appearance: an integrative approach to cancer care should therefore take into consideration the importance of the dimensions of femininity and of the identity of a woman and to help them regain a positive relationship with their body. This paper focuses on this aspect, reporting on the efficacy of an aesthetic care program whose aim is to help women with cancer cope with disease-related changes that can affect their body image and physical appearance during treatment. Unlike more common complementary therapies, few studies have investigated the effect of these types of programs on women's quality of life and global well-being, but the studies that have analyzed this area of research have shown that they might help improve body image, anxiety, self-esteem, and the quality of social interactions (Quintard and Lakdja, 2008; Taggart et al., 2009). Moreover, patients seem to consider these programs as evidence that they are treated as a whole person (Amiel et al., 2009).

The results of this study are in line with the previous literature and with our first study hypothesis (Quintard and Lakdja, 2008; Amiel et al., 2009; Taggart et al., 2009), as they seem to confirm that participating in a psychosocial support program focusing on the aesthetic implications of cancer treatment such as “Health in the Mirror” could determine an improvement in all the psychological variables we chose to evaluate, except quality of life. This might be explained by the complexity of this construct, which may in turn be influenced by multiple variables. Furthermore, the literature shows that it is not uncommon to find relatively good levels of health-related quality of life in cancer patients during chemotherapy treatment (Saevarsdottir et al., 2010), perhaps because patients progressively adapt to their condition, but also because of constant improvements in the management of treatment side effects.

Our results revealed a significant reduction in depressive symptoms, anxiety and body image issues, both immediately at the conclusion of the program and 3 months later, and an improvement in self-esteem levels at the 3-month follow-up; this suggests that participating in our program could facilitate better adjustment to disease and treatment, thus supporting our second study hypothesis. It is possible that a variety of factors could contribute to this result: this program may represent an opportunity for women to regain a positive contact with their bodies, which have been affected by aggressive therapies, and to reconnect with their beauty and femininity. Moreover, getting to know a group of people that share a similar experience may provide these women with an important social support network that prevents isolation.

Further, our research shows that younger women experience more severe symptoms of depression and greater body image issues, as previous studies have already highlighted (Chen et al., 2012; Linden et al., 2012; Simning et al., 2014; Paterson et al., 2016). This result may due to the greater difficulties that younger patients experience while adjusting to a condition of disease, which may have a more profound impact on everyday activities and on their role functioning.

Furthermore, our study revealed that recurrence determines a significant worsening in each variable except anxiety at all time points. Despite the prevalence of recurrent cancer, psychosocial research is still lacking, and the little evidence is conflicting: while some studies found no significant difference in psychological distress and quality of life between patients with primary and recurrent gynecological cancer (Ploos van Amstel et al., 2015), others revealed that in the year following a diagnosis of breast cancer recurrence, women show poorer physical functioning and experience a slower recovery in quality of life compared to newly diagnosed women (Yang et al., 2008). Since our results highlight the negative impact of cancer recurrence on quality of life, depression, body image and self-esteem, more research is needed to further investigate this issue.

An interesting result not reported in the literature that emerged from our research is the negative influence on anxiety and quality of life of diagnoses such as brain, lung, hematologic, and colorectal cancers, when compared to breast cancer, while there seems to be no significant difference between breast and gynecological cancers. This may be due to the fact that often only women affected by these forms of cancer (which are typical of the female gender) are treated in a dedicated unit (for example, Breast Units or Women Cancer Centers) and are included into specific care programs, which promote a comprehensive approach for the patient and her needs. It is possible that women with other diagnoses do not receive the same amount of specific attention, thus feeling more vulnerable; this could in turn determine more intense symptoms of anxiety and a poorer quality of life. These results suggest that patients with these characteristics might represent a risk group that could benefit more from a complementary and supportive program such as “Health in the Mirror.”

In the future, the use of a latent class mixed model (LCMM) approach might be helpful in identifying clusters of patients that may be more vulnerable to psychological distress or benefit more from participation in a psychosocial support program such as “Health in the Mirror.” In addition, it would be interesting to further investigate how being a mother impacts patients' well-being, with a specific focus on the construct of self-esteem; in fact, our study showed that the presence of children seems to have a positive influence on self-esteem, but no study has examined this connection thus far.

We acknowledge several limitations of this research. First, the sample was recruited on a volunteering basis and the characteristics of the patients that declined our invitation are unknown; it is possible that more distressed patients and those in poor physical conditions are not well represented in our sample since they may have rejected participation. Second, results would be strengthened if a control sample was collected concurrently to the Health in the Mirror program; however, there is a difficulty with collecting the control group since knowledge of the existence of the program is rather widespread throughout the hospital and excluding patients from participation to collect a control group would be unethical and unfair. In future research, the collection of a control sample and the examination of potential differences between the control group and the clinical sample might allow us to draw more reliable conclusions on the efficacy of “Health in the Mirror” as a complementary and supportive therapy. Third, there might be an effect of the proximity of the group sessions on some variables at t_1_, such as anxiety levels, which diminish at the conclusion of the program but increase again at t_2_. Finally, even if we identified some interfering variables (for instance, the presence of other stressful life events; one's general medical condition at follow-up; satisfaction for the project) they were not considered to better explain the test scores on anxiety, depression, body image, self-esteem, and quality of life. In the future we will include them in our analysis.

Despite these limitations, this study suggests the importance of implementing complementary and supportive therapies that focus on psychosocial implications of cancer, such as those deriving from treatment-related appearance changes, alongside conventional therapies in hospitals. In fact, the therapeutic approach to cancer cannot be confined only to medical care. In our hospital, the “Health in the Mirror” program is now considered an integral part of cancer treatment as it promotes a better and faster adaptation to the new disease condition. Presently, it represents an example of the “humanization of medical care” in the oncological field (Di Mattei et al., 2015).

## Author contributions

VD was responsible for the conception and design of the research project and the Health in the Mirror project, and approving the final copy. LC was responsible for drafting the work, revising it critically, drafting the study design and she was also involved in data acquisition. MB and PT were responsible for drafting the work, data collection, revising the manuscript and conducting research of the intellectual content. FC and CB were responsible for statistical analyses and data interpretation. AN and MCu were responsible for organizing the project, data collection, and making sure all aesthetic treatments were carried out safely. GM and ER were the oncologists responsible with organizing the project and helping with data collection and proof checking of the manuscript. MCa and LS were responsible for the conception and design of the research project and approving the final copy.

### Conflict of interest statement

The authors declare that the research was conducted in the absence of any commercial or financial relationships that could be construed as a potential conflict of interest.
